# Retraction: ACC-deaminase producing plant growth promoting rhizobacteria and biochar mitigate adverse effects of drought stress on maize growth

**DOI:** 10.1371/journal.pone.0277591

**Published:** 2022-11-16

**Authors:** The PLOS ONE Editors

The *PLOS ONE* Editors retract this article [1, 2] because it was identified as one of a series of submissions for which we have concerns about authorship, competing interests, and peer review. We regret that the issues were not addressed prior to the article’s publication.

SB and MH did not agree with the retraction. MZuH and FM either did not respond directly or could not be reached.

## References

[1] Danish S, Zafar-ul-Hye M, Mohsin F, Hussain M (2020) ACC-deaminase producing plant growth promoting rhizobacteria and biochar mitigate adverse effects of drought stress on maize growth. PLoS ONE 15(4): e0230615. 10.1371/journal.pone.0230615 32251430 PMC7135286

[2] The PLOS ONE Staff (2021) Correction: ACC-deaminase producing plant growth promoting rhizobacteria and biochar mitigate adverse effects of drought stress on maize growth. PLoS ONE 16(4): e0250286. 10.1371/journal.pone.0250286 33844701 PMC8041168

